# Effects of Spartina alterniflora Invasion on Nitrogen Fixation and Phosphorus Solubilization in a Subtropical Marine Mangrove Ecosystem

**DOI:** 10.1128/spectrum.00682-21

**Published:** 2022-05-23

**Authors:** Zufan Zhang, Shiqing Nie, Yimeng Sang, Shuming Mo, Jinhui Li, Muhammad Kashif, Guijiao Su, Bing Yan, Chengjian Jiang

**Affiliations:** a National Engineering Research Center for Non-Food Biorefinery, State Key Laboratory of Non-Food Biomass and Enzyme Technology, Guangxi Key Laboratory of Biorefinery, Guangxi Research Center for Biological Science and Technology, Guangxi Academy of Sciences, Nanning, China; b State Key Laboratory for Conservation and Utilization of Subtropical Agro-Bioresources, Guangxi Research Center for Microbial and Enzyme Engineering Technology, College of Life Science and Technology, Guangxi University, Nanning, China; c Guangxi Key Lab of Mangrove Conservation and Utilization, Guangxi Mangrove Research Center, Guangxi Academy of Sciences, Beihai, China; d State Key Laboratory of Urban and Regional Ecology, Research Center for Eco-Environmental Sciences, Chinese Academy of Sciences, Beijing, China; e University of Chinese Academy of Sciences, Beijing, China; University of Texas at San Antonio

**Keywords:** nitrogen fixation, phosphorus solubilization, shotgun metagenomics, *Spartina alterniflora*, mangrove ecosystem

## Abstract

Nitrogen fixation (NF) and phosphorus solubilization (PS) play a key role in maintaining the stability of mangrove ecosystems. In China, the invasion of Spartina alterniflora has brought a serious threat to the mangrove ecosystem. However, systematic research on NF and PS in mangrove sediments has not been conducted, and limited studies have focused on the response of NF and PS to *S. alterniflora* invasion, particularly at different sediment depths. In the present study, shotgun metagenomics and quantitative PCR were used to study the 0- to 100-cm sediment profile of the mangrove ecosystem in the Beibu Gulf of China. Results showed that the PS potential of mangrove sediments was primarily caused by enzymes encoded by *phoA*, *phoD*, *ppx*, *ppa*, and *gcd* genes. *S. alterniflora* changed environmental factors, such as total nitrogen, total phosphorus, and total organic carbon, and enhanced the potential of NF and PS in sediments. Moreover, most microorganisms involved in NF or PS (NFOPSMs) responded positively to the invasion of *S. alterniflora*. Cd, available iron, and salinity were the key environmental factors that affected the distribution of NF and PS genes (NFPSGs) and NFOPSMs. A strong coupling effect was observed between NF and PS in the mangrove ecosystem. *S. alterniflora* invasion enhanced the coupling of NF and PS and the interaction of microorganisms involved in NF and PS (NFAPSM), thereby promoting the turnover of NP and improving sediment quality. Finally, 108 metagenome-assembled genomes involved in NF or PS were reconstructed to further evaluate NFOPSMs.

**IMPORTANCE** This study revealed the efficient nutrient cycling mechanism of mangroves. Positive coupling effects were observed in sediment quality, NF and PS processes, and NFOPSMs with the invasion of *S. alterniflora*. This research contributed to the understanding of the effects of *S. alterniflora* invasion on the subtropical mangrove ecosystem and provided theoretical guidance for mangrove protection, restoration, and soil management. Additionally, novel NFOPSMs provided a reference for the development of marine biological fertilizers.

## INTRODUCTION

The mangrove ecosystem is a special ecosystem with high productivity and a high nutrient-cycling rate distributed on the tropical and subtropical coastlines, and this ecosystem plays an important role in the biogeochemical cycle (1, 2). However, the invasion of exotic Spartina alterniflora has seriously affected the balance of the mangrove ecosystem (3). Therefore, protection of the mangrove ecosystem is a key scientific issue worldwide. Nitrogen fixation (NF) and phosphorus solubilization (PS) are essential biological processes in sediment, and they play a key role in the restoration and maintenance of mangrove ecosystem stability (4). However, limited studies have determined the effect of *S. alterniflora* invasion on the processes of NF and PS in mangrove sediments. Corresponding countermeasures for mangrove ecosystem protection and soil management can only be implemented after fully understanding the effects of *S. alterniflora* invasion on NF and PS in mangrove sediments.

Although mangrove sediments are limited with nitrogen (N) and phosphorus (P) (5), the mangrove ecosystem can ensure high productivity through effective nutrient cycling (4). NF and PS are essential components of the N and P cycles and are important for the N and P nutrition level of sediments (4). Phosphorus-solubilizing microorganisms (PSMs) in the rhizosphere of mangroves can increase the NF rate of nitrogen-fixing microorganisms (NFMs) (6). Therefore, the coupling effect of NF and PS may also accelerate the turnover of nitrogen and phosphorus (NP). Current reports on the element cycle and biological processes in the mangrove ecosystem have focused on the carbon (C), N, and sulfur (S) cycles and the related biological processes (7, 8). Limited studies have focused on the biological processes related to the P cycle, such as PS, in the mangrove ecosystem. Although many microorganisms involved in NF or PS (NFOPSMs) isolated and cultured from mangrove sediments have been used to develop biological fertilizers (9), a comprehensive understanding of NF, PS, and NFOPSMs in mangrove sediments is lacking. Moreover, a large number of unknown or unculturable functional microorganisms remain in mangrove sediments (8). Thus, these NFOPSMs should be evaluated on a deeper level from microbial communities via metagenomics.

Species invasion is a global concern (10). Exotic *S. alterniflora* has a high growth rate, high salt tolerance, and strong reproductive ability, and its invasion has severely affected the function of the local ecosystem (3). The roots of *S. alterniflora* are primarily distributed in 0- to 30-cm sediments and have occasionally extended to 50 to 100 cm (11). Therefore, the distribution of *S. alterniflora* leads to changes in the physical and chemical properties of sediments at 0 to 100 cm and even deeper, thus affecting the microbial communities and biological processes in the sediments (12). However, most studies have only analyzed the effects of *S. alterniflora* on microbial communities and biological processes in surface sediments (0 to 20 cm) (13, 14). The study of soil profiles at a depth of 100 cm in a salt marsh ecosystem is generally suitable for a comprehensive understanding of biological processes in the soil ecosystem (15). Studying the 0- to 100-cm sediment profile is necessary to evaluate the responses of NF and PS and microorganisms in mangrove sediments to *S. alterniflora* invasion. Therefore, the abundance, activity, and biological processes (such as NF and PS) of microorganisms in mangrove sediments would result in a special response to changes in environmental factors, and the effects of *S. alterniflora* invasion on environmental factors in sediments are sufficient to change the microbial abundance, activity, and potential of NF and PS, thereby mediating NP nutrition and turnover in sediments.

The responses of soil microbial communities and biological processes to *S. alterniflora* invasion have been studied using 16S rRNA gene sequencing and quantitative PCR (qPCR) (13, 14). Nevertheless, 16S universal primers can cause underestimation of the number of specific populations, and the accuracy of qPCR results is usually limited by primers (16, 17). Therefore, metagenomic shotgun sequencing combined with qPCR technology was used to systematically study NF and PS genes (NFPSGs) and NFOPSMs in mangrove sediments. The present study aimed to (i) investigate the relationship among NFPSGs, microorganisms, and physicochemical properties in mangrove sediments; (ii) study the effects of *S. alterniflora* invasion on NFPSGs, microorganisms, and physicochemical properties at different depths; (iii) reveal the coupling mechanism of NF and PS in mangroves and the effects of *S. alterniflora* invasion on the coupling; and (iv) identify potential NFOPSMs.

## RESULTS AND DISCUSSION

### *S. alterniflora* changes the physical and chemical properties in the sediment.

**FIG 1:**
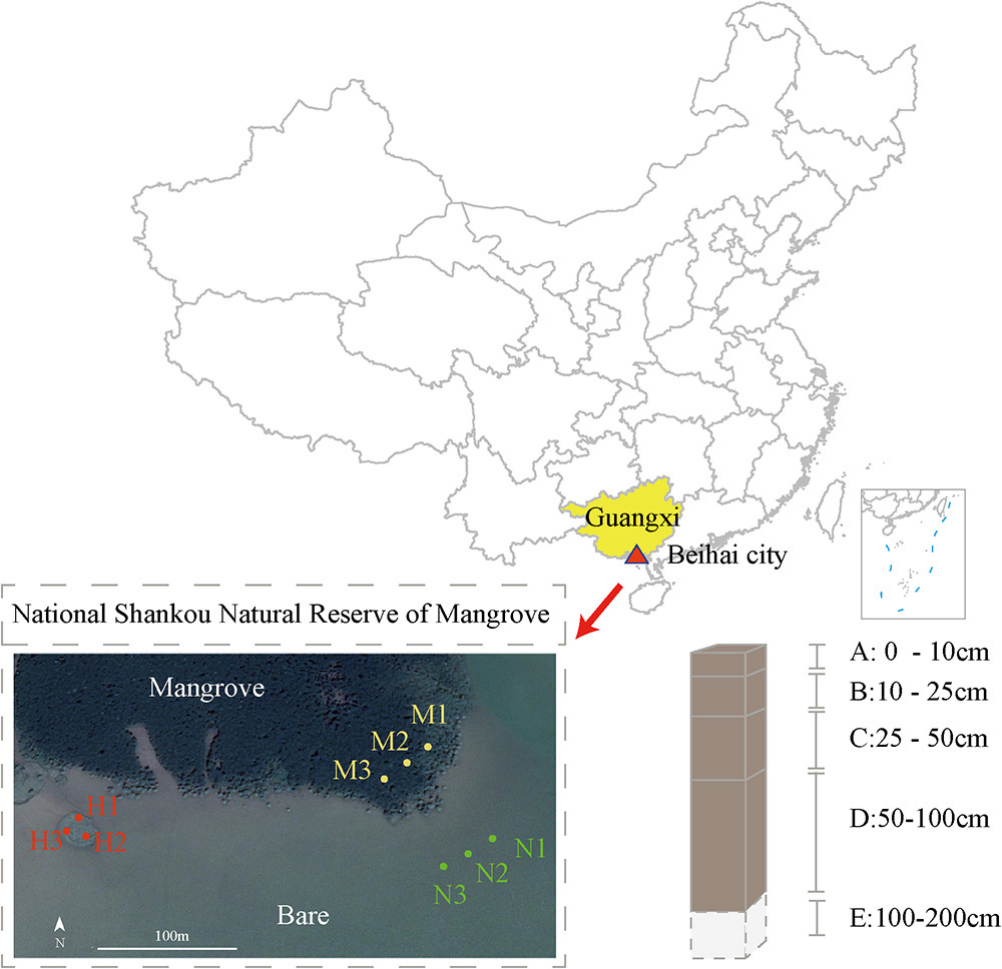
In the sampling map, nine sampling points were obtained from three regions, namely, “M” (*R. stylosa*), “H” (*S. alterniflora*), and “N” (no vegetation). The samples of each sampling point are packed at depths of “A” (0 to 10 cm), “B” (10 to 25 cm), “C” (25 to 50 cm), “D” (50 to 100 cm), and “E” (100 to 200 cm). The geographic information data of the map are from an open database (https://github.com/GuangchuangYu/chinamap/tree/master/inst/extdata/china).

Samples of layer E sediment (100 to 200 cm) were discarded (Fig. 1). Considering the limited number of genes and microorganisms in these samples, the total DNA extracted could not meet the requirements for construction of a shotgun metagenomic sequencing library. At depths of A (0 to 10 cm), B (10 to 25 cm), C (25 to 50 cm), and D (50 to 100 cm) in the H (*S. alterniflora*), M (*Rhizophora stylosa*), and N (bare beach) regions, 36 samples were collected (Fig. 1). The pH, salinity, sulfide, total organic carbon (TOC), total nitrogen (TN), total phosphorus (TP), cadmium (Cd), available iron (AI), and nickel (Ni) were measured to study the differences in physicochemical properties in different regions and correlations among physicochemical properties, NFPSGs, and NFOPSMs (see Table S1A at https://smdb.gxu.edu.cn/filezone/Supplementary_materials.pdf). Mangrove litter and detritus are essential sources of organic carbon in sediments, and organic matter is degraded slowly because of the anoxic environment; therefore, sediments are rich in organic carbon (average TOC content in all samples, 18.01 mg/g; see Table S1A at https://smdb.gxu.edu.cn/filezone/Supplementary_materials.pdf) (18, 19). However, the anoxic and organic-rich characteristics of mangrove sediments improve specific biological processes of microorganisms, such as NF and denitrification (20, 21). Salinity, pH, and temperature are essential for the regulation of microbial abundance in mangrove sediments. In the subtropical mangrove ecosystem, salinity was 26.94 ± 0.25 ppt (mean ± standard error [SE]; *n *= 36), which may provide favorable growth conditions for some salt-tolerant NFOPSMs (see Table S1A at https://smdb.gxu.edu.cn/filezone/Supplementary_materials.pdf) (22, 23). Furthermore, pH regulates mainly soil nutrient availability, and soil pH values between 6.0 and 7.5 are suitable for P availability (13, 24). The pH of all samples was 6.90 ± 0.04 (mean ± SE; *n *= 36), which provide an ideal environment for the microbial community and mangrove plants to obtain the necessary nutrients. Warming can enhance microbial processes, such as NF, in sediments (see Table S1A at https://smdb.gxu.edu.cn/filezone/Supplementary_materials.pdf) (25). However, the temperature was not detected.

Principal-component analysis (PCA), analysis of similarities (ANOSIM), and least significant difference (LSD) were used to show the differences in physical and chemical properties between different regions at each depth (see Fig. S1 and Table S1A at https://smdb.gxu.edu.cn/filezone/Supplementary_materials.pdf). PCA and ANOSIM results showed that the differences in physical and chemical properties were significant among the three groups in B- and D-layer sediments, but no significant differences were observed in the A layer. In addition, significant differences were observed in layer C only between *S. alterniflora* and other areas (*P* < 0.05, see Fig. S1 at https://smdb.gxu.edu.cn/filezone/Supplementary_materials.pdf). The two axes of the PCA explained 60.5%, 58.1%, and 75.5% of the cumulative variance of the physical and chemical properties in layer B, C, and D sediments, respectively (see Fig. S1 at https://smdb.gxu.edu.cn/filezone/Supplementary_materials.pdf). Additionally, N/P, AI, sulfide, and TN of the B layer, TN, TP, salinity, and pH of the C layer, and N/P, TP, TN, and C/P of the D layer had a high total contribution to the first two principal components of the corresponding depth, respectively (see Fig. S1 at https://smdb.gxu.edu.cn/filezone/Supplementary_materials.pdf). Therefore, these physical and chemical properties represented the main differences between samples at corresponding depths. LSD showed that no significant differences in all physical and chemical properties except Ni (H > N, *P* < 0.05) were observed between *S. alterniflora* and other areas (see Table S1A at https://smdb.gxu.edu.cn/filezone/Supplementary_materials.pdf). The TOC, TN, and TP of the corresponding area increases after *S. alterniflora* invades bare beach and sparse mangrove (26). In this study, *S. alterniflora* significantly increased the content of TN of the B layer, TP of the C layer, and TOC, TP, and Cd of the D layer in sediments relative to bare beach (*P* < 0.05, see Table S1A at https://smdb.gxu.edu.cn/filezone/Supplementary_materials.pdf). Therefore, the invasion of *S. alterniflora* may increase the storage capacity of C, N, and P and the accumulation capacity of heavy metals Ni and Cd in the mangrove ecosystem. Considering the NP limitation in mangrove sediments and global warming, the accumulation of these nutrient elements might promote the growth of *S. alterniflora* and enhance its ability to absorb CO_2_ (27, 28). In addition, large differences in physical and chemical properties were observed between *S. alterniflora* and *R. stylosa* area. TN of the B layer, TP and pH of the C layer, and TOC, TP, TN, and Cd of the D layer in sediments of the *S. alterniflora* area were significantly higher than those of the *R. stylosa* area (*P* < 0.05, see Table S1A at https://smdb.gxu.edu.cn/filezone/Supplementary_materials.pdf). However, AI and C/N of the B layer, AI of the C layer, and Ni and C/N of the D layer in sediments of the *S. alterniflora* area were significantly lower than those of the *R. stylosa* area (*P* < 0.05, see Table S1A at https://smdb.gxu.edu.cn/filezone/Supplementary_materials.pdf).

### Main PS strategies in a subtropical marine mangrove ecosystem.

**FIG 2:**
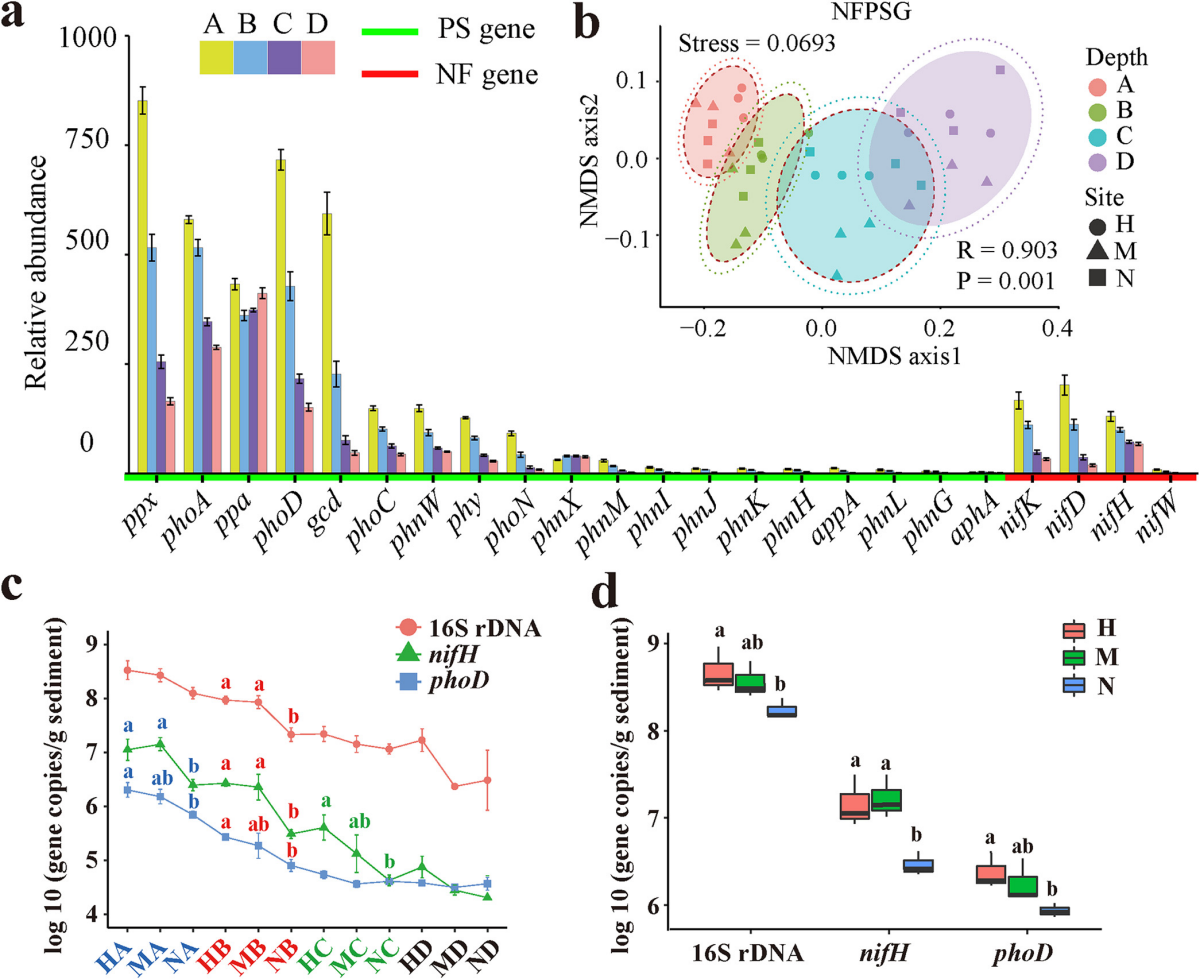
Vertical distribution of NFPSGs and qPCR of key NFPSGs. The SE is expressed as error bars. (a) Relative abundance of NFPSGs at different depths. (b) Nonmetric multidimensional scaling result shows the β diversity of NFPSGs among the groups. The confidence ellipse is generated based on confidence levels of 0.9 and 0.95. The values of *R* and *P* were calculated using ANOSIM. (c) qPCR results of three key genes. Different letters represent significant differences from various areas in each depth (*P* < 0.05, LSD). (d) Average absolute abundance of three key genes in 0- to 100-cm sediments of each area (three sampling points represent three repetitions). Different letters are significantly different from different areas (*P* < 0.05, LSD).

Table S1B (available at https://smdb.gxu.edu.cn/filezone/Supplementary_materials.pdf) shows the read alignment summary of shotgun metagenomic sequencing of 36 samples. A total of 32,961,736 unigenes were obtained by assembly, coding gene prediction, and clustering. Four NF genes and 19 PS genes were annotated using the Kyoto Encyclopedia of Genes and Genomes (KEGG) database (see Table S1C at https://smdb.gxu.edu.cn/filezone/Supplementary_materials.pdf). The top five PS genes were *ppx*, *phoA*, *ppa*, *phoD*, and *gcd* (Fig. 2a; see also Table S1D at https://smdb.gxu.edu.cn/filezone/Supplementary_materials.pdf). Therefore, organic P mineralization involved mainly the hydrolysis of alkaline phosphatase (encoded by *phoA* or *phoD*) to organic phosphoester. Although inositol phosphate (phytate) was the main form of organic P, accounting for up to 50% of the total organic P, the low abundance of phytase genes *phy* and *appA* indicated that most of the organic P was preserved in the form of inositol phosphate, which could not be used by microorganisms and plants (9). Therefore, inorganic P is a large potential pool of soluble reactive P available to plants (9). The production of organic acids by microorganisms is the main solubilization mechanism of inorganic P (24). In addition, gluconic acid is an essential organic acid involved in the solubilization of inorganic P (29). In this study, *gcd*, a gene encoding glucose dehydrogenase, was highly abundant (Fig. 2a; see also Table S1D at https://smdb.gxu.edu.cn/filezone/Supplementary_materials.pdf). Genes encoding inorganic pyrophosphatase (*ppa*) and exopolyphosphatase (*ppx*) were highly abundant, suggesting the absorption of available P and the use of internal poly-P stores (30). These microbial P represent a large amount of immobilized P content, which cannot be used by plants temporarily (31). However, in the long run, all microbial P may be provided to plants. Therefore, the immobilization of microbial P in biomass is an important mechanism for regulating the supply of P in the sediments (31). Moreover, mangroves in the Guangxi province of China are considered a primeval forest far away from any industrial or residential area, so they are less polluted by C-P compounds, such as pesticides, detergents, and additives (32). Nevertheless, the complete core gene (*phnGHIJKLM*) (33) encoding C-P lyase was annotated, indicating the potential to degrade C-P compounds in the mangrove ecosystem (Fig. 2a).

Nonmetric multidimensional scaling (NMDS) analysis and ANOSIM showed significant differences in the vertical distribution of all NFPSGs in sediments (stress = 0.0693 and *P* < 0.05, Fig. 2b). In the mangrove ecosystem, except for *phnX* and *aphA* genes, NFPSGs had the highest relative abundance in layer A sediment (0 to 10 cm), and most of the genes decreased with the increase of depth (Fig. 2a). The *nifH* and *phoD* genes were quantified by qPCR. The results showed that the absolute abundance of genes decreased with increasing sediment depth (*P* < 0.05, Fig. 2c).

### Effects of *S. alterniflora* on NF and PS potential at different sediment depths.

**FIG 3:**
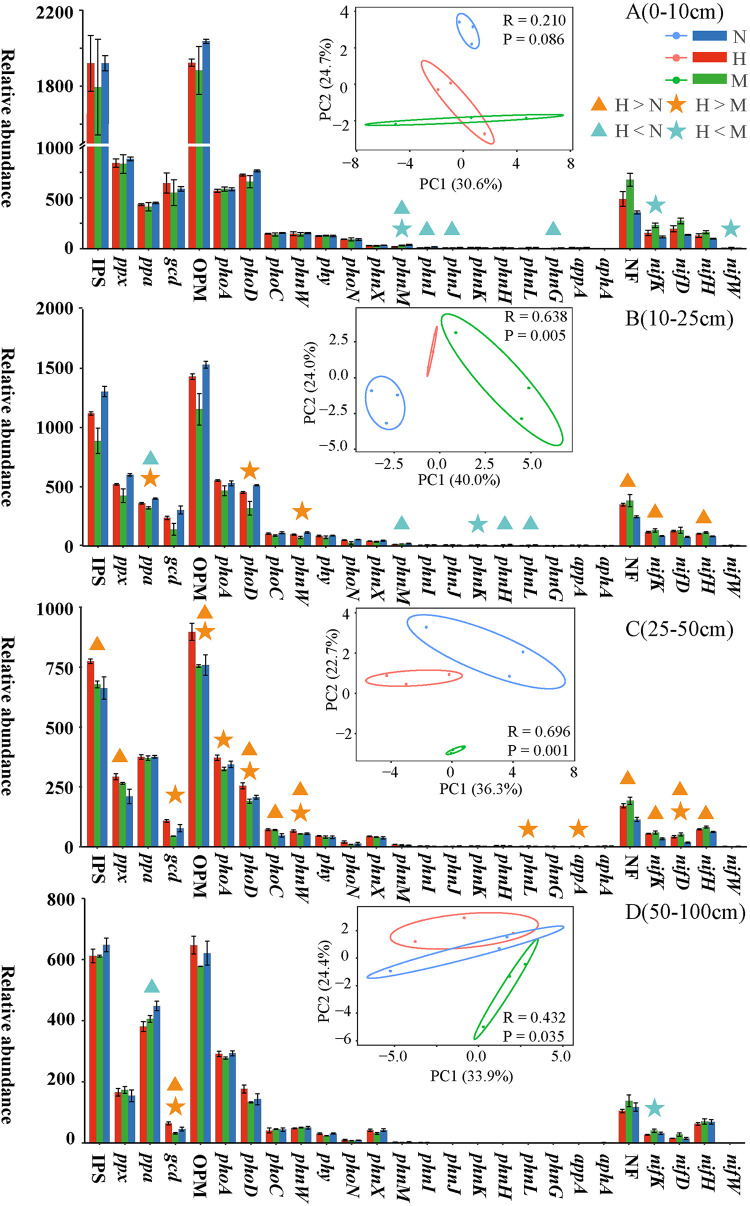
Differences in the distribution of NFPSGs in different areas at each depth. The abundance is the average of each group. SE is expressed as error bars. Nitrogen fixation (NF), inorganic phosphorus solubilization (IPS), and organic phosphate mineralization (OPM) represent the sum of four NF, three IPS, and 16 OPM gene abundances, respectively. The color of the abundance bar represents different regions. Triangles indicate significant (*P < *0.05, LSD) differences between H and N. Stars indicate significant (*P < *0.05, LSD) differences between H and M. PCA plots are of NFPSGs. *R* and *P* were calculated using ANOSIM.

ANOSIM showed significant differences in NFPSGs only in different regions of the B (10 to 25 cm) and C (25 to 50 cm) layers of sediment (*P* < 0.01, Fig. 3). LSD analysis showed that the effect of *S. alterniflora* on NF and PS differed at different sediment depths, and this effect was observed mainly in the C layer (Fig. 3; see also Table S1D at https://smdb.gxu.edu.cn/filezone/Supplementary_materials.pdf). Therefore, similar to the physical and chemical properties (11), the effect of *S. alterniflora* on biological processes in sediments should also be considered in deeper sediment profiles. Consistent with previous studies (13), *S. alterniflora* significantly increased the NF potential in sediment layers B and C after invading bare beach, which is a nonvegetation area (*P* < 0.05, Fig. 3). The qPCR results also showed that the average abundance of *nifH* in the 0- to 100-cm sediments of the *S. alterniflora* area and the absolute abundance of *nifH* in layers A, B, and C of *S. alterniflora* were significantly higher than those in bare beach (*P* < 0.05, Fig. 2c and d). However, at all depths, no significant difference in NF potential was observed between the *S. alterniflora* and native plant (*R. stylosa*) areas. *S. alterniflora* invasion significantly increased the relative abundance of PS genes (*phoC*, *phoD*, *phnW*, and *ppx*) in C-layer sediments (*P* < 0.05), indicating that the increase in PS potential of acid phosphatase (26) and alkaline phosphatase was increased. In B-, C-, and D-layer sediments of the *S. alterniflora* area, the relative abundances of some PS genes (i.e., *ppa*, *phoD*, *phnW*, *phoA*, and *gcd*) were significantly higher than those of *R. stylosa* (*P* < 0.05, Fig. 3). In addition, the average abundance of *phoD* in the 0- to 100-cm sediments and the absolute abundance of *phoD* in A- and B-layer sediments of the *S. alterniflora* area were significantly higher than those in the bare beach area (*P* < 0.05, Fig. 2c and d). However, compared with the metagenomic data, no significant difference in the absolute abundance of *phoD* was observed between the *S. alterniflora* and *R. stylosa* regions (Fig. 2c and d). In summary, *S. alterniflora* increased the potential of NF and PS in sediments, and the potential of NF and PS in the *S. alterniflora* area was equivalent to that of the native plant (*R. stylosa*) area (qPCR data); even the potential of PS in the *S. alterniflora* area was higher than that of the *R. stylosa* area (metagenomics data). A positive correlation was observed between copy number of the NF gene and the rate of NF in sediments (14), and the relative abundance of PS genes was positively correlated with the concentration of P available in soil (29). The first eight genes that determine the concentration of P available in the soil are *gcd*, *phnW*, *phoA*, *ppx*, *phoX*, *phoN*, *olpA*, and *phoD* (29). Therefore, *S. alterniflora* significantly increases the relative abundance of NFPSGs (*nifH*, *nifK*, *nifD*, *phoD*, *phnW*, and *ppx*) and the absolute abundance of key genes (*nifH* and *phoD*) relative to bare beach, which further increases the content of available N and P at the corresponding depth.

### Microorganisms involved in NF or PS in mangrove sediments.

**FIG 4:**
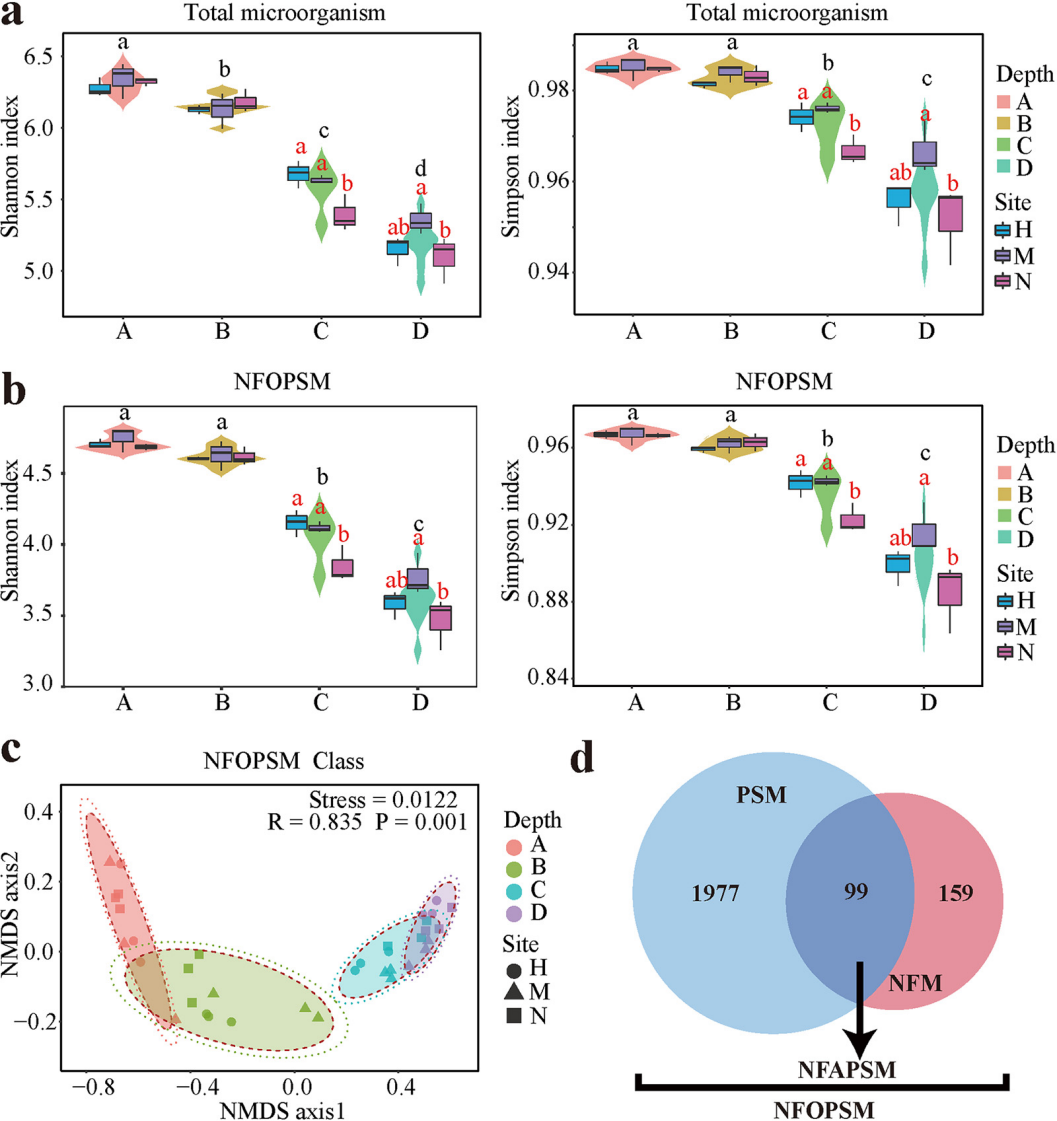
The box plot shows the Shannon and Simpson indices of microorganisms at different depth groups and regions. Different lowercase black letters represent significant differences among depth groups, and different lowercase red letters represent significant differences in different regions (*P* < 0.05). (a) Box plot of the α diversity of all microorganisms. (b) Box plot of the α diversity of NFOPSMs at the class level. (c) Nonmetric multidimensional scaling shows the β diversity of NFOPSMs among the groups. The confidence ellipse is generated based on the confidence levels of 0.9 and 0.95. The values of *R* and *P* were calculated using ANOSIM. (d) Number of species involved in NF and PS.

*Proteobacteria* and *Chloroflexi* were the dominant phyla in mangrove sediments, consistent with the results of Lin et al. (7). *Proteobacteria* were primarily composed of *Gammaproteobacteria*, Deltaproteobacteria, and *Alphaproteobacteria*, while *Chloroflexi* were primarily composed of *Dehalococcoidia* and *Anaerolineae* (see Fig. S2a at https://smdb.gxu.edu.cn/filezone/Supplementary_materials.pdf). A total of 258 species of NFMs and 2,076 species of PSMs were annotated to understand the relationship between microorganisms and NFPSGs in mangrove sediments, among which 99 species were involved in NF and PS (Fig. 4d). Interestingly, NF or PS fungi were not found in the subtropical mangrove sediments. PS fungi species are generally symbiotic with plant roots and are only found under low salinity (<11 ppt) mangrove soils (4); their salt tolerance threshold was 20 ppt (34). The salinity in the mangrove of Beibu Gulf (26.94 ± 0.25 ppt, *n *= 36) was much higher than that of the threshold (see Table S1A at https://smdb.gxu.edu.cn/filezone/Supplementary_materials.pdf). Therefore, colonization of NF or PS fungi rarely exists in the subtropical mangrove sediments. Little or no PS fungi presence has been observed in mangrove ecosystems because of the substitution of PS bacteria for fungi (35, 36). Consistent with Huang et al. (14), classes with a high contribution to NF genes were similar to those with high contribution to PS genes, and *Gammaproteobacteria* and Deltaproteobacteria were the main NFMs and PSMs (see Fig. S3 at https://smdb.gxu.edu.cn/filezone/Supplementary_materials.pdf). The contribution of unclassified species to NFPSGs increases with depth, indicating the presence of a large number of uncultured or unknown NFOPSMs in the deep layer of sediments; thus, the metabolic potential of these functional microorganisms should be explored (see Fig. S3 at https://smdb.gxu.edu.cn/filezone/Supplementary_materials.pdf).

**FIG 5:**
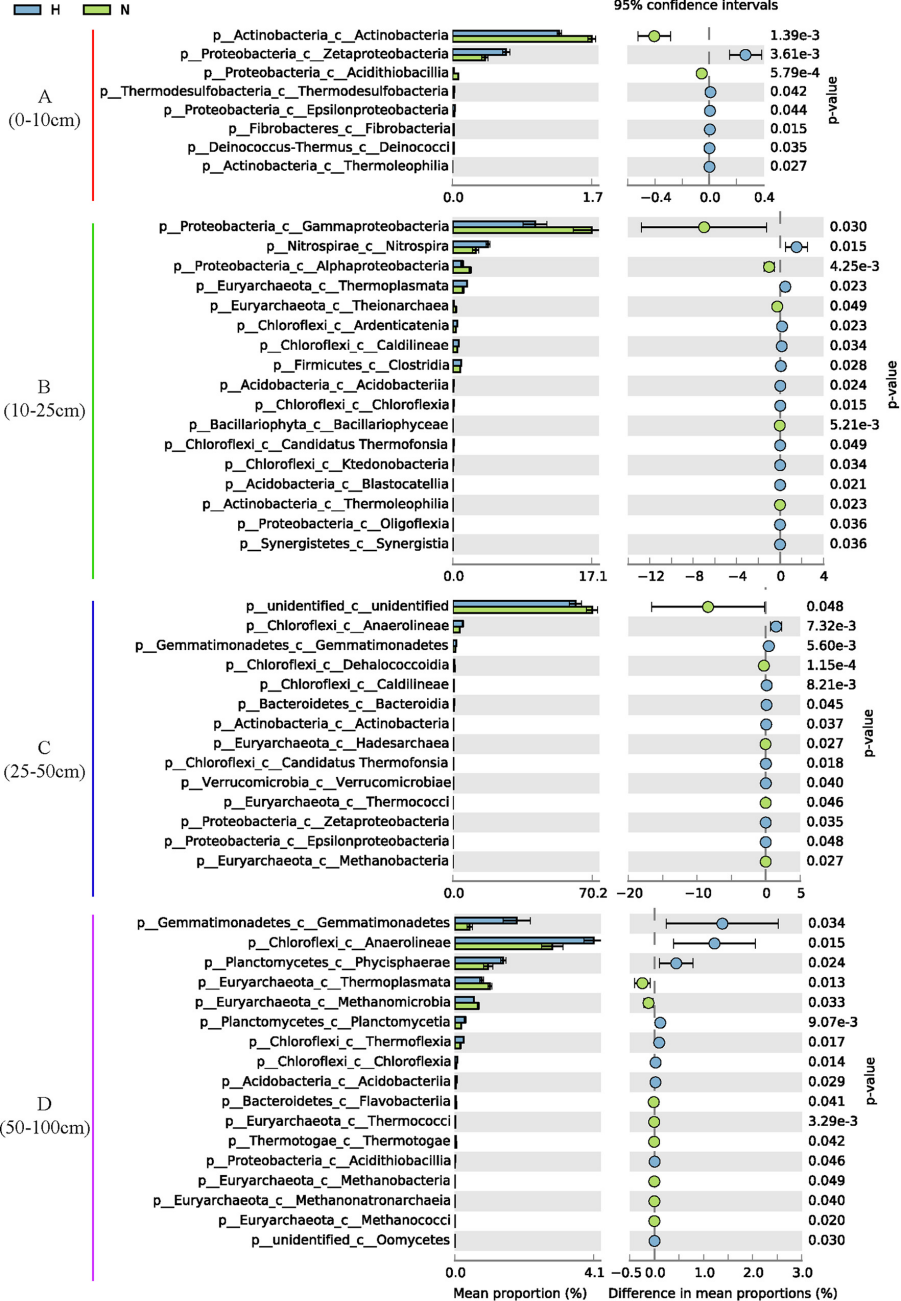
Significant differences of NFOPSMs at the class level between the *S. alterniflora* region and bare beach (*P* < 0.05).

**FIG 6:**
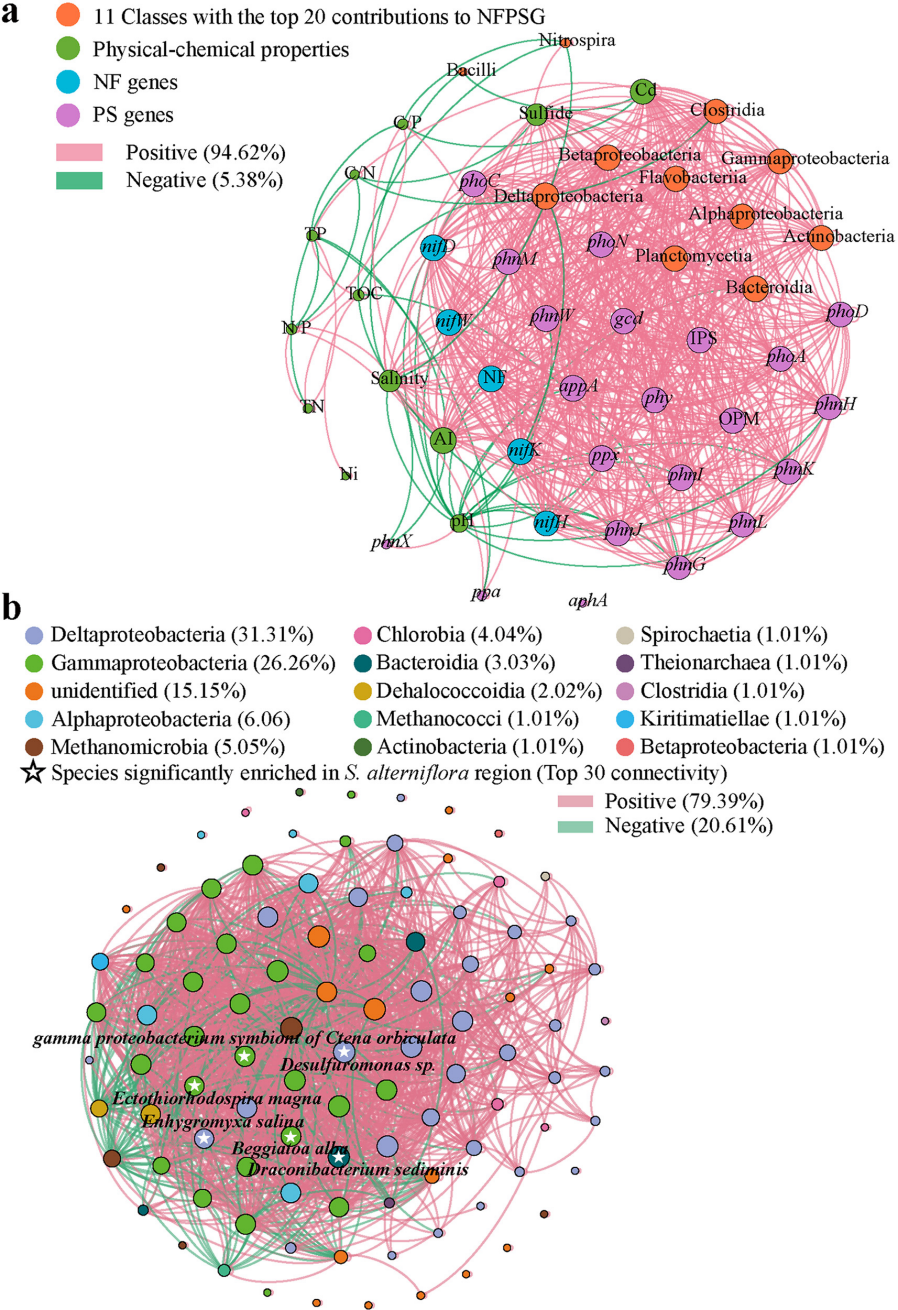
Network diagrams represent Spearman correlations. (a) Spearman correlations between environmental factors, NFPSGs, and 11 classes with the highest contribution to NF genes and PS genes. Edges represent significant Spearman correlations (*P < *0.05 and *|r| *> 0.7, but only *P* < 0.05 for environmental factors). The size of the point represents the degree of connectivity. (b) Correlations among 99 NFAPSMs (*|r| *> 0.8, *P* < 0.05). The color of the point represents the class to which it belongs.

Gao et al. found that *S. alterniflora* invasion significantly reduces the α diversity of soil bacteria (13). In this study, the Shannon and Simpson indices of total microorganisms and NFOPSMs in sediments were calculated. The results showed that *S. alterniflora* invasion significantly increased the α diversity (Shannon and Simpson indices) of total microorganisms and NFOPSMs in C-layer sediments, but no significant effect was observed on other depths (Fig. 4a and b). In addition, no significant difference in the α diversity of total microorganisms and NFOPSMs was found between the *S. alterniflora* and *R. stylosa* regions (Fig. 4a and b). The qPCR results of 16S rRNA gene sequencing showed that the invasion of *S. alterniflora* increased the total bacterial abundance in the sediment (*P* < 0.05, Fig. 2c and d). NMDS and ANOSIM results showed that NFOPSMs significantly differ at different depths (stress = 0.0122 and *P* < 0.05, Fig. 4c). PCA results indicated significant differences in different regions of the B, C, and D layers among NFOPSMs at the class level (*P* < 0.05, see Fig. S2b at https://smdb.gxu.edu.cn/filezone/Supplementary_materials.pdf). Figure 5 shows that in the A, B, C, and D layers of the *S. alterniflora* region, 6, 12, 9, and 9 classes were significantly higher than those in bare beach, respectively, while 2, 5, 4, and 8 classes were significantly lower than those in bare beach, respectively (*P* < 0.05). Therefore, the invasion of *S. alterniflora* notably influenced NFOPSMs at the class level, and most of the NFOPSMs (such as classes belonging to *Chloroflexi*) had a positive response to the invasion of *S. alterniflora*. Interestingly, most of the classes belonging to *Euryarchaeota* in the *S. alterniflora* region were significantly lower than those in bare beach, especially in the D layer (Fig. 5). Spearman correlation analysis showed that most NFOPSMs had a strong positive correlation with NFPSGs (Fig. 6a). Therefore, the positive response of NFOPSMs to *S. alterniflora* invasion at the class level was beneficial to NF and PS in the *S. alterniflora* region. The average contribution of *Gammaproteobacteria* to NFPSGs in the B layer of *S. alterniflora* were less than those in bare beach because of the significant reduction of the abundance of *Gammaproteobacteria* (*P* < 0.05, see Fig. S3 and S5 at https://smdb.gxu.edu.cn/filezone/Supplementary_materials.pdf). The invasion of *S. alterniflora* significantly increased the contribution of Deltaproteobacteria to NF genes in B- and C-layer sediments (*P* < 0.05, see Fig. S3 at https://smdb.gxu.edu.cn/filezone/Supplementary_materials.pdf). Therefore, Deltaproteobacteria provided more NF genes in the B and C layers. However, no significant difference in Deltaproteobacteria abundance was observed between the *S. alterniflora* and bare beach regions (Fig. 5), possibly because of the increased activity of Deltaproteobacteria, which provided more copies of NF genes (37). In addition, the contribution and abundance of major PSMs (Deltaproteobacteria and *Gammaproteobacteria*) to PS genes in the *S. alterniflora* region were significantly different from those in the *R. stylosa* region (*P* < 0.05, see Fig. S3 and S4a at https://smdb.gxu.edu.cn/filezone/Supplementary_materials.pdf).

### Key environmental factors and the coupling of NF and PS.

In this study, Cd, AI, and salinity were found to be the key environmental factors affecting the distribution of NFPSGs and NFOPSMs (Fig. 6). The correlations of pH and sulfide with NFPSGs and NFOPSMs were second only to these key environmental factors (Fig. 6). Cd, salinity, AI, and sulfide were positively correlated, whereas pH was negatively correlated, with NFPSGs and NFOPSMs (Fig. 6). Increased salinity causes the elimination of salt-intolerant species (38). However, within the appropriate salinity range, the abundance of salt-tolerant microorganisms (e.g., *Proteobacteria*) will increase with increasing salinity (38). Therefore, the general salt tolerance of NFOPSMs existed in subtropical mangrove ecosystems. In the mangrove ecosystem, NFOPSMs include many sulfate-reducing bacteria (4). In an anoxic environment, these NFOPSMs could reduce SO_4_^2−^ to S^2−^ and reduce iron to a form that is not conducive to P binding while fixing N and solubilizing P (4). In addition, heavy metal ions could combine with S^2−^ to accumulate heavy metals and sulfides in sediments (4, 39). This evidence demonstrates that the coupling between the C, N, P, S, and iron cycles is mediated by NFOPSMs in sediments. However, when a large amount of SO_4_^2−^ is reduced and these heavy metals exceed a certain threshold, a certain toxicity to these NFOPSMs is introduced (4, 39). Therefore, the differences in physical and chemical properties between the *S. alterniflora* region and other regions directly or indirectly affect the distribution and activity of NFOPSMs and the potential of NF and PS. Although high TN, TP, TOC, and potential of NF and PS were found in the *S. alterniflora* area, these physicochemical properties were not significantly correlated with NFPSGs and NFOPSMs (Fig. 6). The physicochemical properties of sediments are often regulated by microorganisms as well as plants and animals (40). Similarly, the abundance and function of microorganisms are affected by various environmental factors. The roots of *S. alterniflora* are distributed mainly in the first 30 cm of the sediment and sometimes extend to 50 to 100 cm underground, resulting in different effects on the physical and chemical properties of different depths (11). Benthos and algae also affect physical and chemical properties (41). Moreover, tidal inundation, wave action, plant roots, and biological disturbance are essential factors for determining the microbial abundance below 60 cm in mangrove sediments (23).

In mangrove sediments, a strong coupling between the cycling of elements in C, N, S, and P, particularly the coupling of NP, is important for NP nutrition (7, 42). The addition of N in sediments, such as ammonia N through nitrogen deposition/biological NF, can enhance the ability of plants to absorb P in sediments, increase the yield of litter, and accelerate the decomposition rate of organic matter, mineralization rate of the net N and P, and nutrient release (25, 43). NF in sediments is an indispensable source of N in the mangrove ecosystem (4), and PSMs can promote the NF of NFMs (6). Therefore, the coupling of NF and PS mediated by NFOPSMs in sediment promotes the turnover of NP in the mangrove ecosystem. Twenty-three NFPSGs were used for Spearman correlation analysis. Strong positive correlations (212 positive correlations and 0 negative correlations) among NFPSGs were observed in the mangrove ecosystem (Fig. 6a). Thus, coupling between NF and PS is conducive to the turnover of NP in mangrove sediments. Zhang et al. (44) found that a small amount of inorganic P could enhance the mineralization ability of bacteria to phytic acid. In the present study, inorganic PS genes (*ppx* and *gcd*) were positively correlated with phytase genes (*phy* and *appA*) and other phosphatase genes, indicating a possible strong coupling effect among different PS modes (Fig. 6a). A total of 215, 210, and 209 positive correlations of 23 NFPSGs were observed in *S. alterniflora*, *R. stylosa*, and bare beach regions, respectively. Considering the high potential of NF and PS in the *S. alterniflora* region, the NFPSGs in this region had a stronger coupling.

The combined inoculation of PSMs and NFMs could improve their viability. In addition, they could produce hormones that promote plant root growth and use insoluble P by a synergistic effect (45). Therefore, the interaction of NFOPSMs can enhance the utilization of NP nutrition and improve the growth and yield parameters of various plants. Considering that the interaction of microorganisms involved in NF and PS (NFAPSMs) drives the coupling of NF and PS, 99 NFAPSMs were used for Spearman correlation analysis. These NFAPSMs belonged to mainly Deltaproteobacteria (31.31%) and *Gammaproteobacteria* (26.26%). A total of 1,499 correlations were recorded among NFAPSMs, among which 1,190 correlations were positive (Fig. 6b). The network topology characteristic parameters included an average path length of 1.65, average clustering coefficient of 0.83, and modularity of 0.35 (values of <0.4 suggest the absence of modular structure). The results of modularity indicated that the difference in NFAPSM function in the mangrove ecosystem was not enough to form a modular structure, whereas the result of average clustering coefficient indicted that NFAPSMs have complex and strong interactions. Therefore, NFAPSMs exist in the mangrove ecosystem as a class of community with the same or similar functions (such as NF and PS). In addition, this condition led to the change of individual nodes (NFAPSMs), especially the nodes with high connectivity degree, thus remarkably affecting the whole network. These NFAPSMs had 1,561, 1,555, and 1,315 positive correlations in the *S. alterniflora*, bare beach, and *R. stylosa* regions, respectively. Among the NFAPSMs with a top 30 connectivity degree, 6, 2, and 0 were significantly enriched in the *S. alterniflora*, bare beach, and *R. stylosa* regions, respectively, as determined by linear discriminant analysis (LDA) effect size (LEfSe) analysis (Fig. 6b). Therefore, the invasion of *S. alterniflora* may positively affect NFAPSMs and their interactions in sediments, thus enhancing the turnover of NP nutrition.

### Novel NFOPSMs.

**FIG 7:**
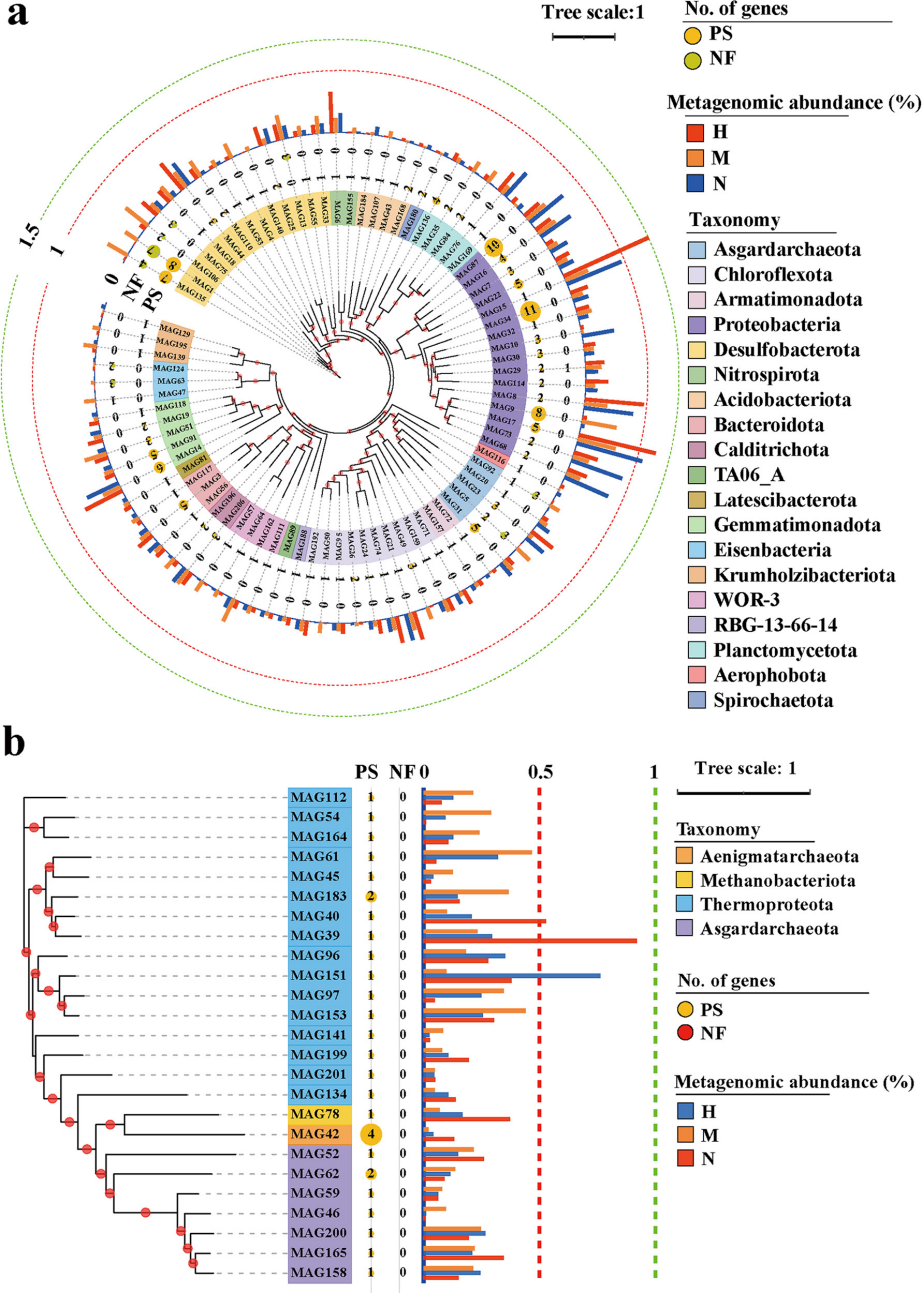
Maximum likelihood phylogenetic tree. Bootstrap values were based on 1,000 replicates, and percentages higher than 50% are shown in red circles. The numbers of genes encoding enzymes that are responsible for PS or NF identified in MAGs are shown in the middle. The outermost layer shows the relative abundance of MAGs in H, M, and N. (a) Maximum likelihood tree of 83 bacterial MAGs. (b) Maximum likelihood tree of 25 archaeal MAGs.

A total of 207 metagenome-assembled genomes (MAGs; completeness ≥ 70%, contamination ≤ 10%) were obtained in these MAGs to explore new NFOPSMs in sediments (https://doi.org/10.6084/m9.figshare.13611557.v2). A total of 108 MAGs (including 83 bacteria and 25 archaea) were involved in NF or PS (see Table S1E at https://smdb.gxu.edu.cn/filezone/Supplementary_materials.pdf). In addition, 5 (MAG1, 5, 29, 92, and 135) of the 108 MAGs were involved in NF and PS, indicating that these MAGs are important nutrient bacteria in sediments as well as potential drivers of NP coupling (Fig. 7; see Table S1E at https://smdb.gxu.edu.cn/filezone/Supplementary_materials.pdf). Moreover, some MAGs with high relative abundance (such as MAG13 and MAG17) and high number of NF genes or PS genes (such as MAG13 and MAG34) are essential for the sediment NP nutrient level (Fig. 7; see Table S1E at https://smdb.gxu.edu.cn/filezone/Supplementary_materials.pdf). MAG bacteria were primarily composed of *Proteobacteria* (16), *Desulfobacterota* (14), and *Chloroflexota* (10), and MAG archaea were primarily composed of *Thermoproteota* (16) and *Asgardarchaeota* (7). Only 44 MAGs were annotated to the genus level, and no MAGs were annotated to the species level, indicating that many MAGs belong to novel NFOPSMs, and many unknown microorganisms in the sediment are involved in NF or PS. These MAGs were primarily involved in PS through alkaline phosphatase (encoded by *phoA* and *phoD*), exopolyphosphatase (*ppx*), inorganic pyrophosphatase (*ppa*), and glucose dehydrogenase (*gcd*) (see Fig. S4b at https://smdb.gxu.edu.cn/filezone/Supplementary_materials.pdf). An individual MAG (MAG87) was annotated to the complete core gene (*phnGHIJKLM*) of C-P lyase. Therefore, this strain could degrade C-P compounds. Six MAGs (MAG1, 5, 13, 75, 92, and 135) had relatively complete nitrogenase genes (*nifHDK*), indicating their importance for N input in sediments. Among these MAGs, MAG1 and MAG135 have relatively complete nitrogenase genes (*nifHDK*) and various PS genes, thus providing reference for the development of marine biological fertilizers (45).

LEfSe analysis showed that 27 of the 108 MAGs were significantly enriched in *S. alterniflora*. In the sediments of the A, B, C, and D layers, 2, 5, 19, and 8 MAGs were significantly enriched in the *S. alterniflora* area, respectively (see Table S1E at https://smdb.gxu.edu.cn/filezone/Supplementary_materials.pdf). Less MAGs were enriched in the A- and B-layer sediments of *S. alterniflora* than in the two other areas, whereas the same number of MAGs was enriched in the C-layer sediments of the *S. alterniflora* and *R. stylosa* areas, but a higher number was enriched than that observed in bare beach. Furthermore, more MAGs were enriched in the layer D sediments of the *S. alterniflora* area than in *R. stylosa* and bare beach areas (see Table S1E at https://smdb.gxu.edu.cn/filezone/Supplementary_materials.pdf). The enrichment of these MAGs in *S. alterniflora* sediments contributed to NP turnover.

### Conclusion.

NF and PS of the subtropical mangrove ecosystem in Beibu Gulf of China were systematically investigated. The results revealed the efficient nutrient cycling mechanism of mangroves and provided a theoretical basis for mangrove protection, restoration, and soil management with *S. alterniflora* invasion. The responses of NF, PS, and NFOPSMs in sediments to the invasion of *S. alterniflora* were conducive to the further invasion of *S. alterniflora*. Therefore, after invading bare beach, prompt and strict control of *S. alterniflora* is necessary to prevent further invasion in the mangrove vegetation coverage area. Finally, the novel NFOPSMs (MAG1 and MAG135) with relatively complete nitrogenase genes and diverse PS genes provide new insight into the development of marine biological fertilizers.

## MATERIALS AND METHODS

### Sampling sites.

The National Shankou Natural Reserve of Mangrove is located in the subtropics; it has a total coastline of 50 km and a total area of 8,000 km^2^. Twelve species of mangrove plants were identified in the reserve, including *R. stylosa* and *Avicennia marina*. The alien invasive species *S. alterniflora* was also found in the reserve. In November 2019, 36 samples were collected in three areas of the reserve (21°29′25.74″N, 109°45′49.43″E), including the *R. stylosa* cover area (M), *S. alterniflora* cover area (H), and nonvegetation area (N) (Fig. 1). Three sampling sites were randomly selected in each region (10 m × 10 m), and visible litter and stones were removed. Five 0- to 200-cm sediment cores were collected using a stainless steel sector soil collector with a diameter of 10 cm and three 50-cm stainless steel connecting pipes at each sampling site with a sample square of 0.5 m × 0.5 m. Each core was cut by using a 13-cm aseptic plastic shovel into 0 to 10 cm, 10 to 25 cm, 25 to 50 cm, 50 to 100 cm, and 100 to 200 cm. Then, the sediment cores of the same depth at each sampling point were thoroughly mixed in a sterile bag as a sample. The samples from the *R. stylosa* region were marked “M1,” “M2,” and “M3.” The samples from the *S. alterniflora* region were marked as “H1,” “H2,” and “H3.” The samples from the nonvegetation area were marked “N1,” “N2,” and “N3.” The samples obtained from the depths of 0 to 10, 10 to 25, 25 to 50, 50 to 100, and 100 to 200 cm were named “A,” “B,” “C,” “D,” and “E,” respectively. Finally, all the samples were transported back to the laboratory on the same day by using a well-sealed ice box (0°C). Each sample was divided into three parts for shotgun sequencing, physical and chemical property determination, and qPCR and refrigerated at −20°C until further processing.

### Physicochemical analysis.

Sediments were centrifuged to obtain sediment suspensions, and the pH and salinity of sediment suspensions were determined using a pH meter (PHS-2C, China Sanxin) and salinity meter (PAL-06S, Atago, Japan), respectively. TP and sulfide were determined by a colorimetric procedure (46). Total nitrogen (TN) was determined using an automatic carbon and nitrogen analyzer (TOC-TN 1200, Thermo Euroglas), and the TOC was determined after carbonate removal by washing with 10% HCl. The AI concentration was determined using an atomic absorption spectrophotometer (GBC932, Australia) according to the manufacturer’s instructions. Samples were treated by HNO_3_/H_2_O_2_ digestion (47), the supernatant was filtered through a 0.45-μm filter membrane, and the concentrations of nickel (Ni) and cadmium (Cd) in sediments were determined by inductively coupled plasma mass spectrometry (PerkinElmer Corp., Norwalk, CT).

### DNA extraction and sequencing.

The FastDNA spin kit for soil (MP Biomedicals, USA) was used to extract total DNA from 0.5 g of sediments. DNA quality and quantity were detected by 1% agarose gel electrophoresis and analysis with a NanoDrop 2000 spectrophotometer (Thermo Scientific, USA). According to a previous method (48), at least 5 μg of total DNA was sent to GENEWIZ, Inc. (Beijing, China), for next-generation sequencing library preparation and sequencing. Next-generation sequencing libraries were prepared following the manufacturer’s protocol (VAHTS Universal DNA library prep kit for Illumina). Then, libraries with different indices were multiplexed and loaded onto an Illumina HiSeq instrument according to the manufacturer’s instructions (Illumina, San Diego, CA, USA).

### Metagenomic analysis.

Appropriate modifications were made for metagenomic analysis as previously described (49). FastQC v0.11.9 (50) was used to check the quality of the sequencing results. Raw shotgun sequencing reads were trimmed using cutadapt v3.2 (51). Primers, adapters, and bases with a mass value of less than 20 at both ends and reads with an N base content of more than 10% were removed, and the minimum read length of 75 bp was retained. Clean data were assembled using MEGAHIT v1.2.9 to obtain contigs (>500 bp) by using the default parameters (52). SoapAligner v2.21 (53) was used to obtain unused reads for mixed assembly. The comparison parameters were -u, -2, and -m 200. These reads were assembled as described above. The genes of each sample were predicted using Prodigal v3.02 (54). CD-hit v4.8.1 (55) was used to cluster genes derived from all samples with a default identity of 0.95 and coverage of 0.9. Paired-end clean reads were mapped to unigenes by using SOAPAligner to generate read coverage information for unigenes and analyze the relative abundance of unigenes in each sample. Gene abundance was calculated based on the number of aligned reads and normalized to gene length. Finally, all samples were normalized based on the sequencing depth of the smallest sample to obtain the relative abundance of unigenes.

### Taxonomic classification, functional annotation, and genome binning.

BLASTP of diamond v0.9.32 (56) was used to search the protein sequences of the unigenes in the NCBI nonredundant (nr) and KEGG databases. The statistical significance threshold of the sequence alignment was set to 1 × 10^−5^. The least common ancestor (LCA) algorithm of MEGAN v6 (57) was used for the taxonomic assignment. In addition, the sequence alignment length was set to no less than 60% of the reference gene protein length. Resulting matches with the best scores were selected for functional annotation. The species corresponding to NF genes were defined as NFMs, while those corresponding to PS genes were defined as PSMs (Fig. 4d). The union and intersection of NFMs and PSMs were defined as NFOPSMs and NFAPSMs, respectively (Fig. 4d). The contribution rate of microorganisms to NF or PS was calculated as previously described (8). The relative abundances of NFPSGs were calculated by summing the relative abundances of unigenes annotated to the NFPSGs. The relative abundances of taxonomical ranks were calculated by summing the relative abundances of unigenes annotated to the taxonomical rank. Then, the α diversity values of all microorganisms and NFOPSMs were calculated based on the relative abundances of microorganisms at the species level, respectively.

Contigs with lengths of <1,500 bp were removed. The remaining contigs were binned using MetaBAT2 v2.12 (58), MaxBin2 v2.2 (59), and CONCOCT v1.0 (60) with default parameters to obtain the MAGs. dRep v1.4.3 (61) was used to cluster MAGs at the species level with an identity of 0.95. CheckM v1.1.2 (62) was used to select high-quality MAGs with integrity higher than 70% and pollution lower than 10% for further analysis. The abundance of contigs was calculated as the method of gene abundance calculation, and the abundance of each MAG was calculated as the average contig abundance. Then, the relative abundance of each MAG in each sample was determined by dividing the abundance of each MAG with the total abundance of all MAGs in each sample. The contigs of MAGs were predicted and functionally annotated as described above. MAGs containing NF or PS genes were selected for further analysis. GTDB-Tk v2.1.15 (63) was used to infer the taxonomic classifications of the selected MAGs and to construct two phylogenetic trees of bacterial MAGs and archaeal MAGs based on 120 bacterial and 122 archaeal conserved marker genes. The phylogenetic tree was visualized on the iTOL website (https://itol.embl.de).

### qPCR.

The nitrogenase gene (*nifH*), alkaline phosphatase gene (*phoD*), and bacterial 16S rRNA gene were quantified using the fluorescent dye SYBR green approach on a Roche LightCycler 480 II with primer sets nifHF/nifHRb (64), ALPS-F730/ALPS-R1101 (65), and 341f/797r (66), respectively. The plasmid standard (pMD 18murt vector), which carries the corresponding gene, was diluted to 1 ng/μL and then diluted in a gradient 10 times. Approximately 1 μL of each gradient was used for the standard curve generation. All amplification correlation coefficients (*R*^2^) were greater than 0.99. During qPCR detection, each sample was diluted 5 or 10 times according to the concentration, and then 1 μL of the diluent DNA was collected as the reaction quantity. Each sample was tested three times. Details about the primer sets and the experimental procedures are summarized in Table S1F (available at https://smdb.gxu.edu.cn/filezone/Supplementary_materials.pdf).

### Statistical analysis.

The α diversity of microorganisms (Shannon and Simpson indices) was calculated using the R packet “vegan.” The LSD test was used based on the analysis of variance (ANOVA) model for multiple comparisons among our treatments groups for physicochemical characteristics, functional genes, contribution rate of microorganisms to NF or PS, and α diversity of NFOPSMs. The ANOVA model and the LSD test were calculated using the R packet “stats” and “agricolae.” A PCA was used to describe the differences of physicochemical characteristics among samples from different regions by using the R package “stats.” NMDS and ANOSIM were used to depict the differences in Bray-Curtis distances at a 0.05 significance level among different groups based on the relative abundance of NFOPSMs and NFPSGs by using the R package “vegan.” A Welch’s *t* test and LEfSe (http://huttenhower.org/galaxy) were used to estimate the significantly enriched NFOPSMs and MAGs in each group, respectively. The α value for the factorial Kruskal-Wallis test among classes and pairwise Wilcoxon test among subclasses was set to 0.05. In addition, the threshold of the logarithmic LDA score for discriminative features was set to 2. A Spearman correlation analysis was used to correlate the relative abundances of NFPSGs and NFOPSMs with the content of physical and chemical properties and to investigate the correlation of NFPSGs and the interactions of NFAPSMs by using the R package “psych.” *P* values were corrected using the Benjamini-Hochberg method. The significant Spearman correlations were filtered according to the following parameters: significant Spearman correlations between environmental factors, NFPSGs, and NFOPSMs (*P < *0.05 and *|r| *> 0.7, but only *P* < 0.05 for environmental factors) and significant Spearman correlations among 99 NFAPSMs (*|r| *> 0.8, *P* < 0.05). The correlation between NFPSGs and the correlation between NFAPSMs in each region were calculated based on 12 samples of the corresponding region. The network based on significant Spearman correlation was generated, and the network topology characteristic parameters, including average path length, average clustering coefficient, and modularity were calculated using Gephi software.

### Data availability.

The metagenomic data sets have been deposited at the Chinese National Genomics Data Center GSA database (https://ngdc.cncb.ac.cn/) under BioProject number PRJCA002729. The 207 MAGs are saved in figshare (https://doi.org/10.6084/m9.figshare.13611557.v2).
